# Upregulation of GBP1 in thyroid primordium is required for developmental thyroid morphogenesis

**DOI:** 10.1038/s41436-021-01237-3

**Published:** 2021-06-30

**Authors:** Rui-Meng Yang, Ming Zhan, Qin-Yi Zhou, Xiao-Ping Ye, Feng-Yao Wu, Mei Dong, Feng Sun, Ya Fang, Rui-Jia Zhang, Chang-Run Zhang, Liu Yang, Miao-Miao Guo, Jun-Xiu Zhang, Jun Liang, Feng Cheng, Wei Liu, Bing Han, Yi Zhou, Shuang-Xia Zhao, Huai-Dong Song

**Affiliations:** 1 The Core Laboratory in Medical Center of Clinical Research, Department of Molecular Diagnostics & Endocrinology, Shanghai Ninth People’s Hospital, State Key Laboratory of Medical Genomics, Shanghai Jiao Tong University School of Medicine, Shanghai, China; 2 Department of Urology, Shanghai Ninth People’s Hospital, Shanghai Jiao Tong University School of Medicine, Shanghai, China; 3 Department of Head and Neck Surgery, Renji Hospital, Shanghai Jiao Tong University School of Medicine, Shanghai, China; 4 Department of Endocrinology, Maternal and Child Health Institute of Bozhou, Bozhou, China; 5 Department of Endocrinology, The Central Hospital of Xuzhou Affiliated to Xuzhou Medical College, Xuzhou, China; 6 Department of Laboratory Medicine, Fujian Provincial Maternity and Children’s Hospital, Fuzhou, China; 7 Stem Cell Program, Boston Children’s Hospital and Harvard Stem Cell Institute, Boston, MA USA; 8 Division of Hematology/Oncology, Boston Children’s Hospital and Dana Farber Cancer Institute, Boston, MA USA

## Abstract

**Purpose:**

Congenital hypothyroidism (CH) is a common congenital endocrine disorder in humans. CH-related diseases such as athyreosis, thyroid ectopy, and hypoplasia are primarily caused by dysgenic thyroid development. However, the underlying molecular mechanisms remain unknown.

**Methods:**

To identify novel CH candidate genes, 192 CH patients were enrolled, and target sequencing of 21 known CH-related genes was performed. The remaining 98 CH patients carrying no known genes were subjected to exome sequencing (ES). The functions of the identified variants were confirmed using thyroid epithelial cells in vitro and in zebrafish model organisms in vivo.

**Results:**

Four pathogenic *GBP1* variations from three patients were identified. In zebrafish embryos, *gbp1* knockdown caused defective thyroid primordium morphogenesis and hypothyroidism. The thyroid cells were stuck together and failed to dissociate from each other to form individual follicles in *gbp1*-deficient embryos. Furthermore, defects were restored with wild-type human *GBP1* (*hGBP1*) messenger RNA (mRNA) except for mutated *hGBP1* (p.H150Y, p.L187P) overexpression. GBP1 promoted β-catenin translocation into the cytosol and suppressed the formation of cellular adhesion complexes. Suppression of cell–cell adhesion restored the thyroid primordium growth defect observed in *gbp1*-deficient zebrafish embryos.

**Conclusion:**

This study provides further understanding regarding thyroid development and shows that defective cellular remodeling could cause congenital hypothyroidism.

## INTRODUCTION

Congenital hypothyroidism (CH) is the most common inherited endocrine disorder, affecting approximately 1 in 3,000–4,000 newborns worldwide [1–4]. Untreated congenital hypothyroidism inevitably leads to cretinism, causing irreversible brain dysfunction and dwarfism. The majority of CH diseases are caused by thyroid dysgenesis (TD) in the White population, which may manifest as athyreosis, thyroid ectopy, hypoplasia, or hemiagenesis [5, 6]. These diseases are due to embryonic thyroid development defects. The molecular mechanisms underlying TD are unknown. The four transcription factors NKX2-1, FOXE1, PAX8, and HHEX are considered fundamental to thyroid formation [7–9]. However, the pathogenic variants in these genes only explain a minority of TD patients, suggesting that unknown genetic and epigenetic factors might play important roles in thyroid development [10, 11].

Tissue morphogenesis involved several processes that shape tissues and organisms and relies on the coordinated regulation of cell behavior [12]. Adherent junctions (AJs) are protein complexes found at cell–cell junctions of epithelial and endothelial tissues that connect the actin cytoskeleton of adjacent cells [13]. From bud to the gland, thyroid morphogenesis is a multistage process [14]. Before bilobation and fusion with the ultimobranchial body (UBB), all migrating thyroid bud progenitor cells stick together as a single body [15], and E-cadherin expression is maintained throughout this process [16]. However, immediately after entering the thyroid bud and lose contact with the pharyngeal cavity, the tight junction protein ZO-1 is downregulated [17] and does not reappear until follicles are formed [18]. Therefore, cellular architectures are highly variable and should be dynamically regulated to obtain the formation of an integral thyroid gland. Either enhanced or weakened cellular connections would be detrimental to TP remodeling. Understanding the internal regulatory mechanisms might help us identify TD causative genes.

In this study, exome sequencing (ES) was performed on 98 unrelated CH patients, all of whom have been excluded from carrying the 21 known CH candidate genes [19], to identify novel causal genes for CH. Four pathogenic *GBP1* variations were identified in three patients in three pedigrees, all of whom were functionally impaired. Using a zebrafish model and thyroid cell line for in vivo and in vitro studies, we demonstrated that GBP1 plays an indispensable role in regulating embryonic thyroid primordium (TP) morphogenesis to form functional thyroxine-producing units. We also found that this is mediated by its role in triggering β-catenin-associated adhesion complex degradation.

## MATERIALS AND METHODS

### Clinical subject enrolment and exome sequencing

Neonates with CH were screened based on thyroid-stimulating hormone (TSH) levels. This was performed using filter paper blood spots (obtained through heel prick) obtained 3–5 days after birth. The diagnostic criteria for establishing CH in patients have been described previously [19–21]. This study included unrelated infants with CH (*n* = 192). ES data processing and variant calling were performed as described in Supplementary Methods. Written consent was obtained from the parents of patients, and the study was approved by the Ethics Committee of Shanghai Ninth People’s Hospital affiliated with the Shanghai Jiao Tong University School of Medicine. The 57 normal thyroid tissues were collected from patients with thyroid benign nodules according to pathological diagnosis from Shanghai Renji Hospital affiliated with the Shanghai Jiao Tong University School of Medicine. Written consent was obtained from the patients, and the study was approved by the Ethics Committee of Shanghai Renji Hospital affiliated with the Shanghai Jiao Tong University School of Medicine.

### Zebrafish husbandry

Zebrafish maintenance and staging were performed as previously described [22]. The zebrafish facility and study were approved by the Institutional Review Board of the Institute of Health Sciences, Shanghai Institutes of Biological Sciences, Chinese Academy of Sciences (Shanghai, China). Animals were randomly assigned to each group. No animals were excluded from the study and all animals were allocated to groups based on genotype. Detailed materials were described in the Supplementary Methods.

### Cell culture and reagents

Cells were cultured in a humidified incubator at 37 °C in the presence of 5% CO_2_. Detailed methods for immunofluorescence assay and western blots were described in the Supplementary Methods.

### Statistical analysis

Data were presented as mean ± standard error of mean (SEM). Group comparisons of normally distributed data were performed with unpaired Student’s *t*-test. SPSS17.0 software (IBM, Chicago, IL, USA) was used for all statistical analyses. Statistical significance was taken to be *P* < 0.05.

## RESULTS

### *GBP1* variations identified from patients with congenital hypothyroidism

**Fig. 1:**
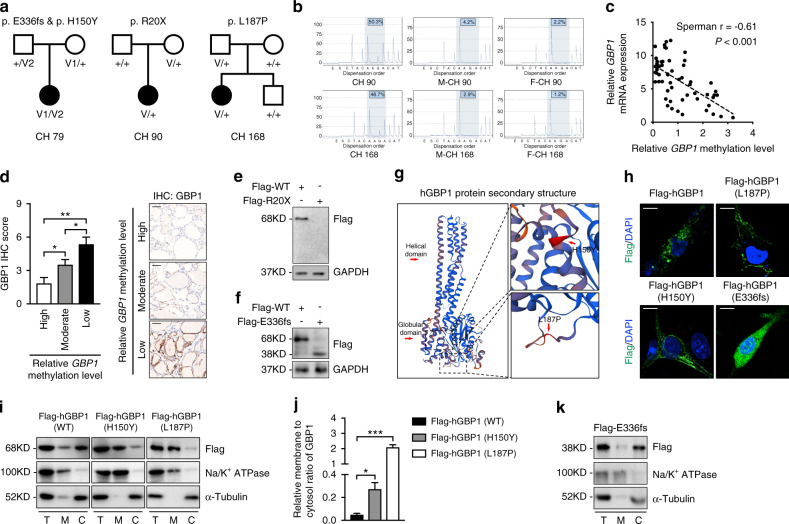
Identification of *GBP1* variations in congenital hypothyroidism (CH) patients. (**a**) Transmission pattern of *GBP1* variations in three pedigrees. Proband 79 (CH 79) carried compound heterozygous variations (p.H150Y and p.E336fs) inherited from the euthyroid father and mother respectively. Proband 90 (CH 90) carried heterozygous variation inherited from the euthyroid mother. Proband 168 (CH 168) carried a heterozygous variation (p.L187P). V variation, black solid box: proband. (**b**) The cg12054698 methylation status examined in CH 90, CH 168, and their parents by pyrosequencing. F father, M mother. Relative methylation level of the CpG site of each sample was labeled. (**c**) The correlation of cg12054698 methylation status with the messenger RNA (mRNA) levels of human *GBP1* (*hGBP1*) in 57 normal thyroid tissues (Spearman *r* = −0.61, *P* < 0.001). (**d**) Left is the quantification of GBP1 expression by immunohistochemistry (IHC) classified by cg12054698 methylation level assessed by methylation-specific polymerase chain reaction (PCR). Right is the representative IHC staining of GBP1 in cg12054698 high, moderate, and low methylated thyroid tissues. Bars = 50 μm. (**e**) Western blot examination of the R20X mutated GBP1 expression in TPC1 cells. (**f**) Western blot confirmation of the truncated GBP1 caused by p.E336fs variation in TPC1 cells. (**g**) The position of the two missense variations of *hGBP1* (H150Y and L187P) in the secondary structure of hGBP1 protein. (**h**) The cellular location of transfected *Flag-hGBP1*, *Flag-hGBP1-H150Y*, *Flag-hGBP1-L187P*, and *Flag-hGBP1-E336fs* in TPC1 cells by immunofluorescence assay. Scale bar: 10 μm. (**i**) The expression of Flag-hGBP1, Flag-hGBP1-H150Y, and Flag-hGBP1-L187P in the cell membrane and cytosol compartment detected by western blot. Na/K^+^ ATPase was used as a loading control for the membrane and α-tubulin for the cytosol fraction. (**j**) The ratios of hGBP1 expression in the membrane to that in the cytosol, calculated by gray value scan in three independent experiments. (**k**) Membrane cytosol extraction show the content of the mutated hGBP1 (p.E336fs) in each compartment. Na/K^+^ ATPase was used as a loading control for the membrane and α-tubulin for the cytosol fraction. Means ± SEM are shown for three independent experiments. *P < 0.05; **P < 0.01; ***P < 0.001; (Student’s *t*-test).

To identify novel CH candidate genes, 192 CH patients were enrolled, and target sequencing of 21 known CH-related genes was performed [19]. The remaining 98 patients with CH carrying no known genes were subjected to ES. Four pathogenic *GBP1* variations were identified in three patients from three pedigrees (Fig. 1a). Thyroid dysgenesis was found in two patients, and diffuse hypoechoic mass, which indicated internal structural abnormalities of the thyroid, was observed in all three patients by B-ultrasound (Table 1). Furthermore, the transmission patterns of *GBP1* in the three pedigrees were investigated using Sanger sequencing. This study found that the proband (CH 79) in pedigree 79 carried compound heterozygous *GBP1* variations (Fig. 1a). However, CH 90 and CH 168 had a heterozygous *GBP1* variation inherited from either their euthyroid mother or father (Fig. 1a). Therefore, the heterozygous *GBP1* variation did not explain the pathogenesis of CH in pedigrees 90 and 168. Although phenotypes are mainly driven by genetic variants, accumulating evidence supports that epigenetic mechanisms also contribute to mammalian interindividual phenotypic differences [23]. An open sea differentially methylated region (DMR) in the 5′UTR of *GBP1* was found to be hypomethylated in triple-negative breast cancer (TNBC) samples [24]. Using methylation-specific polymerase chain reaction (PCR), this study found that the CpG site (cg12054698) of *GBP1* was hypermethylated in the genomic DNA isolated from probands CH 90 and CH 168 compared with their euthyroid parents, who carried the same variation (Fig. S1A and S1B). To quantitatively determine the methylation status of the CpG sites, pyrosequencing of this region was carried out. Consistently, hypermethylation levels of the CpG site (cg12054698) were found in the two patients (Fig. 1b). No obvious differences in the total DNA methylation level were found between the affected children and their parents, as calculated using the 5-mC DNA ELISA kit (Fig. S1C). Methylation levels of the CpG site (cg12054698) were also examined in ten affected children of the same district, matched for age and sex of CH 90 and CH 168 by pyrosequencing (Fig. S1D). However, methylation at cg12054698 maintained at low level among all these samples examined (Fig. S1D). To determine the relationship between the methylation status and the expression level of GBP1, 57 normal thyroid tissues were collected. The relationships between the methylation level of the CpG site (cg12054698), calculated by methylation-specific PCR, and the expression level of GBP1, calculated by either quantitative PCR (qPCR) or immunohistochemistry, were examined. An inverse relationship between the methylation levels of cg12054698 and the expression of GBP1 were found (Fig. 1c and d). Therefore, the results suggest that both genetic and epigenetic factors contribute to the penetration of CH in the two pedigrees.

**Table 1:** Clinical information of the three congenital hypothyroidism (CH) patients carrying *GBP1* variations in our study.

ID	Age at onset (day)	FT3 (2.5–3.9) pg/mL^a^	FT4 (0.58–1.64) ng/dl^b^	TSH (0.34–5.6) uIU/ml	Thyroid ultrasound
CH 79	20	2.81	NA	144	Dysgenesis with diffuse hypoecho
CH 90	15	NA	<0.4	169	Normal size with diffuse hypoecho
CH 168	15	NA	NA	124	Dysgenesis with diffuse hypoecho

*NA* not available, *TSH* thyroid-stimulating hormone.

^a^FT3: free tri-iodothyronine. Conventional unit is used here. International System of Units (SI) = conventional unit × 1.54 pmol/L.

^b^FT4: free thyroxine. Conventional unit is used here. International System of Units (SI) = conventional unit × 12.87 pmol/L.

GBP1 harbors an N-terminal global GTPase domain and a C-terminal α-helix domain required for dimerization. The latter is subdivided into the middle domain and C-terminal α12/13 domain [25–27]. We constructed plasmids of mutant and wild-type (WT) *hGBP1* with Flag fused in the N-terminal and transfected them into human papillary thyroid carcinoma TPC1 cells. The premature stop codon at the 20th amino acid (p.R20X) led to no protein generation (Fig. 1e), and the frameshift variation (p.E336fs) led to the generation of a truncated GBP1 as detected by western blotting (Fig. 1f). Both missense variants (p.H150Y and p.L187P) were located near the region of contact between the LG domain and the α12/13 domain of hGBP1 (Fig. 1g). Disruption in this region has been reported to result in hGBP1 localization to the membrane [28–30]. The influences of the two missense variants (p.H150Y and p.L187P) on GBP1 localization in cells were detected by immunofluorescence (IF) and western blotting. Consistent with previous reports, WT hGBP1 was mostly located in the cytoplasm structures such as the Golgi complex (Fig. 1h). In contrast to WT hGBP1, the two mutated GBP1 (p.H150Y and p.L187P) preferred to stabilize on the membrane (Fig. 1h). The frameshift variation (p.E336fs) hGBP1 was diffusely expressed in the cytosol (Fig. 1h). By extracting proteins from the cell membrane and cytosol of TPC1 cells, this study confirmed the preferred location of the two mutated hGBP1s (p.H150Y and p.L187P) on the cell membrane by western blotting (Fig. 1i and j). Consistent with the IF results, GBP1 with the frameshift variation (p.E336fs) was mainly expressed in the cytosolic fraction (Fig. 1k). Accordingly to the American College of Medical Genetics and Genomics/Association for Molecular Pathology (ACMG/AMP) criteria, all variants were classified as likely pathogenic or pathogenic respectively (Table S1) [31].

### *Gbp1* deficiency in zebrafish led to abnormal thyroid development

Molecules involved in thyroid development are essentially conserved between zebrafish and mammals [32]. In zebrafish, the corresponding time for thyroid fate specification from the foregut endoderm in the pharyngeal floor at approximately 24 hours postfertilization (hpf). The growing midline TP buds off from the pharyngeal floor from 24 to 26 hpf and moves to the outflow of the heart at 48 hpf [33, 34]. From 48 hpf, the TP extends caudally near the anterior neck region, accompanied by folliculogenesis and terminal differentiation of progenitor cells to hormone-producing thyrocytes that express thyroglobulin (*tg*) and sodium iodide symporter (*nis*) [35]. Through whole-mount in situ hybridization (WISH) staining of *tg* expression, we observed that after relocating the cardiac outflow at 48 hpf, the surface areas of TP gradually increased near the midline of the ventral pharyngeal area (Fig. S2A and S2B). The morphology of the growing TP is characterized by one front point in the anterior and two bilateral stripes in the posterior region. The angle between the two posterior stripes reduced gradually to fuse into a single strand at approximately 5 days postfertilization (dpf) during thyroid development (Fig. S2C).

**Fig. 2:**
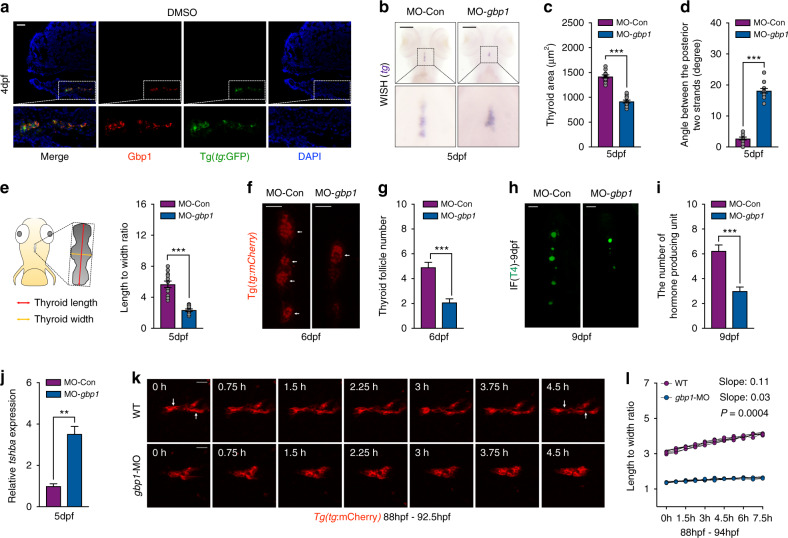
*Gbp1* knockdown led to hypothyroidism and thyroid developmental abnormalities in zebrafish embryos. (**a**) Representative image showing the expression of Gbp1 in 4-dpf thyroid tissue under Tg(*tg*:GFP) background using immunofluorescence (IF) assay. (**b**) Whole-mount in situ hybridization (WISH) assessment of *tg* expression in *gbp1* morphants at 5 dpf. (**c**–**e**) Statistical calculation of thyroid area (**c**), angle between the posterior strands (**d**), and length to width ratio (**e**) in *gbp1* morphants. *N* = 12. Left schematic illustration in (**e**) showing how the length and width of the zebrafish thyroid primordium (TP) is calculated. While the length is measured from the front point to the ending point of the TP along the pharyngeal midline, the width is the measured by the widest axis vertical to the length axis. (**f**, **g**) Representative image (**f**) and statistical analysis (**g**) of follicle formed with *gbp1* knockdown under Tg(*tg*:mCherry) background. *N* = 12. Bars = 20 μm. (**h**,**i**) Representative image (**h**) and statistical analysis (**i**) of T4 producing units with *gbp1* knockdown. *N* = 12. Bars = 20 μm. (**j**) *Tshba* transcripts detected in 5-dpf wild-type (WT) and *gbp1* morphants. Means ± SEM are shown for three independent experiments. (**k**) Representative images chosen from Movie S1 and Movie S2 showing the differential growth mode of TP in 88 hpf−92.5 hpf WT and *gbp1* deficient embryos. Bars = 20 μm. (**l**) Statistical calculation of the dynamic changes of the length to width ratio in WT and *gbp1* morphants from 88 to 94 hpf. *N* = 3. The arrows indicate the separation of follicles after 4.5 h morphogenesis. **P < 0.01; ***P < 0.001 (Student’s *t*-test).

The zebrafish genome contains one annotated *gbp1* with 45% amino acid homology with hGBP1 (Fig. S3A). Similar to hGBP1, zebrafish Gbp1 (zfGbp1) also contains an N-terminal globe GTPase domain and a C-terminal helix domain (Fig. S3B). Gbp1 was highly expressed in the thyroid gland at 4 dpf zebrafish embryos by IF assay performed on frozen sections (Fig. 2a). A *zfgbp1* knockdown in zebrafish was conducted using a morpholino designed against the translation initiation site of *zfgbp1*. The knockdown efficiency was determined by western blot assays (Fig. S3C). Early primordium specification and follicular differentiation of the thyroid in zebrafish seemed undisturbed after *zfgbp1* knockdown. This is due to the expression of *nkx2.1a* and *pax2.1a*, two key transcription factors involved in early thyroid development, were normally expressed at 26 hpf (data not shown). However, at 5 dpf, in contrast with the loose organization of TP along the pharyngeal midline in the WT control embryos by *tg* staining, condensed thyroid tissue was observed in *gbp1* morphants (Fig. 2b). As the TP surface area decreased, the angles measured between the two posterior stripes were larger, and the stripe length to width ratios were reduced in *gbp1* deficient embryos (Fig. 2c–e). The reduction in follicle numbers (Fig. 2f and g) and thyroxine-containing units (Fig. 2h and i) support the hypothesis that hypothyroidism develops with *gbp1* deficiency. *Tshba* transcripts, one of the most sensitive markers for hypothyroidism diagnosis, were increased in 5-dpf *gbp1*-deficient embryos compared with WT controls, as detected by reverse transcription PCR (RT-PCR) (Fig. 2j).

Through time-lapse observation of TP growth in vivo, we observed that from 3.5 dpf, the TP elongation along the midline accompanies cluster separation, facilitating the formation of individual follicles (Movie S1 and Fig. 2k). However, thyroid cells stick together, and in situ proliferation without successful separation are the main features of TP growth with *gbp1* deficiency (Movie S2 and Fig. 2k). The length to width ratio of TP gradually increased with time in WT embryos from 88 hpf to 94 hpf. However, it maintained a low value in *gbp1-*deficient embryos (Fig. 2l). The late thyroid remodeling process near the midline is in close contact with the surrounding vascular cells. However, using Tg(*flk:*GFP) and Tg(*tg:*mCherry) double transgenic lines, we found that the vascular structure around the thyroid tissue was otherwise normally developed after *zfgbp1* knockdown, suggesting that disturbed thyroid cell migration did not result from abnormal vascular development (Fig. S4A). Through pH3 and TUNEL staining, we found that apoptotic *tg*-positive cells were increased in *gbp1*-deficient embryos at 3 dpf (Fig. S4B). However, thyroid cell proliferation remained relatively unchanged (Fig. S4C).

### Functional impairment of mutated *GBP1* identified in patients

**Fig. 3:**
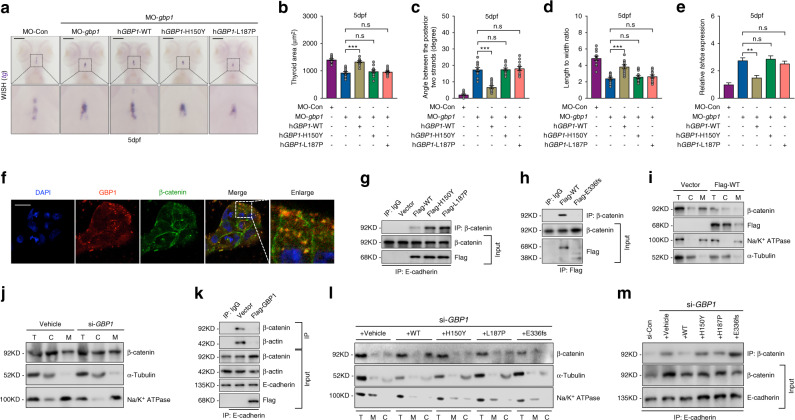
Functional impairment of *GBP1* variants identified in congenital hypothyroidism (CH) patients. (**a**) The rescuing effects of co-expression of wild-type (WT) *hGBP1* or mutated *hGBP1* messenger RNA (mRNA) (*hGBP1-H150Y* or *hGBP1-L187P*) on thyroid development are displayed. Bars = 50 μm. (**b**–**d**) Statistical analysis of the thyroid area (**b**), angle between two posterior strands (**c**), and length to width ratio (**d**) in WT and *gbp1* morphants with WT or mutated *hGBP1* mRNA overexpression. *N* = 12. (**e**) Rescuing effects *tshba* expression in *zfgbp1* morphants with WT or mutated *hGBP1* mRNA overexpression. Means ± SEM are shown for three independent experiments. (**f**) The expression of endogenous hGBP1 and β-catenin in TPC1 cells was detected by immunofluorescence. Bars = 20 μm. The enlarged image shows that hGBP1 colocalized with β-catenin in the cytoplasm of TPC1 cells. (**g**) Co-immunoprecipitation (Co-IP) assessment of the interaction between WT and mutated hGBP1 (p.H150Y, p.L187P) with β-catenin. (**h**) Co-IP assessment of the interaction between the truncated hGBP1 (p.E336fs) with β-catenin. (**i**) Western blot examination of cytosol and membrane β-catenin content with overexpression of N-terminal flag fused hGBP1. Na/K^+^ ATPase was used as a loading control for the membrane and α-tubulin for the cytosol fraction. (**j**) The effect of *GBP1* knockdown in TPC1 cells on the content of cytosol and membrane β-catenin. Na/K^+^ ATPase was used as a loading control for the membrane and α-tubulin for the cytosol fraction. (**k**) The effect of introducing N-terminal flag fused GBP1 on the formation of adhesion complex in TPC1 cells. (**l**) Restoring effects of WT or mutated *hGBP1* overexpression on the subcellular β-catenin levels in *GBP1*-deficient TPC1 cells. Na/K^+^ ATPase was used as a loading control for the membrane and α-tubulin for the cytosol fraction. (**m**) Rescuing effects of WT or mutated *hGBP1* overexpression on cellular adhesion complex formation in *GBP1*-deficient TPC1 cells. Means ± SEM are shown for three independent experiments. n.s. not significant; **P < 0.01; ***P < 0.001 (Student’s *t*-test).

We then examined the rescuing effects of the mutated *GBP1* using a zebrafish model organism. The highly adhesive TP morphology caused by *gbp1* deficiency was specifically restored by combined WT *hGBP1* messenger RNA (mRNA) overexpression. However, no rescuing effects were observed with the two mutated *hGBP1* (p.H150Y, p.L187P) overexpression (Fig. 3a–d). Additionally, *tshba* transcripts were efficiently reduced with WT *hGBP1* mRNA overexpression, suggesting a restored thyroid function. Similar rescue effects were not observed in the two mutated *hGBP1* (Fig. 3e). This study also found that thyroid cells overexpressing *zfgbp1*, fulfilled by cloning *zfgbp1* under the *tg* promoter, showed an increased propensity for distant migration along the endothelia (Fig. S5A). Ectopic *tg-*positive thyroid cells were also detected by WISH after embryos overexpressing *zfgbp1* in thyroid cells (Fig. S5B). To ensure that migratory behavior was induced by *hGBP1*, we constructed TPC1 cells stably overexpressing WT *hGBP1* (WT-TPC1). Compared with TPC1 cells transfected with an empty vector, migration was faster for *hGBP1*-TPC1, as assessed by a scratch assay (Fig. S5C). Additionally, the expression of migration-related proteins such as vimentin (VIM), IQGAP1, and MMP2 was found to be upregulated in WT-TPC1 cells (Fig. S5D).

### GBP1 retains β-catenin in the cytosol and suppressed cellular adhesion complex formation

AJs are dynamically regulated during cellular invasion or migration, modifying their composition to stimulate some of the signaling pathways and cytoskeleton organization involved in cellular motility [36]. Catenin protein connects E-cadherin to the actin cytoskeleton, which leads to the formation of epithelial cell–cell adhesions. In TPC1 cells, this study found that endogenous hGBP1 colocalized well with cytosolic β-catenin (Fig. 3f). Their interactions were confirmed by co-immunoprecipitation (Fig. 3g). WT hGBP1 interacted with β-catenin, and this capacity was similar to that of the two hGBP1 mutants (H150Y and L187P) (Fig. 3h). Truncated GBP1 failed to interact with β-catenin (Fig. 3h).

This study investigated whether GBP1 could influence the cellular location of β-catenin. By extracting the membrane and cytosol components, it was found that WT *hGBP1* overexpression promoted cytosolic β-catenin content (Fig. 3i), whereas *GBP1* knockdown had the opposite effect, suggesting that GBP1 promoted the retention of β-catenin in the cytoplasm (Fig. 3j and Fig. S5E). In addition, *GBP1* overexpression weakened cell–cell adhesion as the amount of β-catenin and F-actin interacting with E-cadherin was reduced (Fig. 3k). WT *hGBP1* overexpression restored the reduced cytosol/plasm content of β-catenin caused by *GBP1* deficiency except for the three mutated GBP1 (p.H150Y, p.L187P, and p.E336fs) (Fig. 3l). The formation of cell–cell adhesion complexes in TPC1 cells was also examined. Cellular connections were increased with *GBP1* knockdown as increased β-catenin was pooled down by E-cadherin, which was restored with WT *hGBP1* overexpression (Fig. 3m). No similar restoring effects were observed with the three mutated *hGBP1* (Fig. 3m), confirming functional impairment of all three mutated *GBP1* identified in CH patients.

### Suppression adhesion complex formation restored thyroid development abnormalities in *gbp1* morphants

**Fig. 4:**
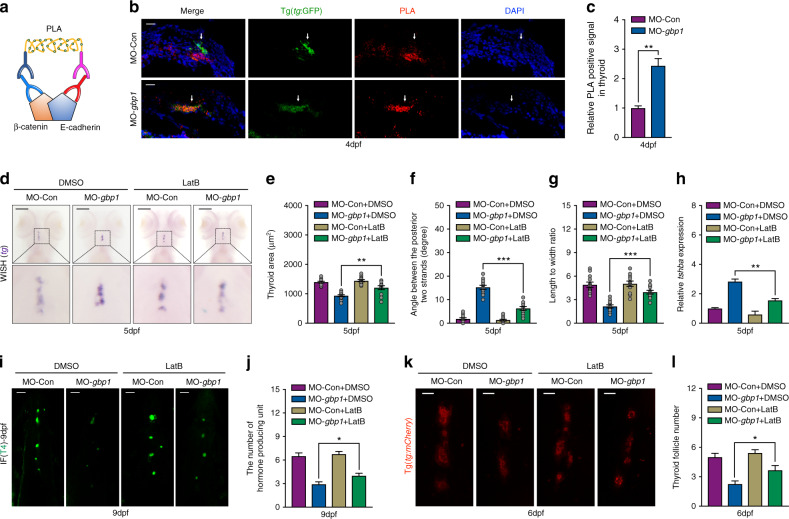
Cellular adhesion inhibition restored the abnormal thyroid primordium (TP) morphology in *gbp1-*deficient embryos. (**a**) Cartoon image show the working model of Duolink PLA strategy. (**b**,**c**) Representative images (**b**) and statistical assessment (**c**) of the β-catenin and E-cadherin interaction pattern in 4-dpf wild-type (WT) and *gbp1* deficient embryos under Tg(*tg*:GFP) background. Means ± SEM of the three independent experiments are shown. (**d**–**g**) Representative image showing *tg* expression pattern (**d**), statistical analysis of the thyroid area (**e**), angle between two posterior strands (**f**), and length to width ratio (**g**) in WT and *gbp1* morphants with or without LatB addition. *N* = 12. (**h**) Restoring effects on *tshba* expression in WT and *gbp1* morphants with or without LatB addition. Means ± SEM of the three independent experiments are shown. (**i**,**j**) Representative image (**i**) and statistical analysis (**j**) showing the rescuing effects of LatB treatment on follicle number in *gbp1* morphants under Tg(*tg*:mCherry) transgenic background. *N* = 12. Bars = 20 μm. (**k**,**l**) Representative image (**k**) and statistical analysis (**l**) showing the rescuing effects of LatB treatment on T4 containing units in *gbp1* morphants by immunofluorescence (IF) assay. *P < 0.05; **P < 0.01; ***P < 0.001 (Student’s *t*-test).

The inefficient cell dissociation of TP was the main feature observed with *gbp1* morphants, and GBP1 overexpression inhibited cellular adhesion complex formation. Therefore, this study examined the formation of cell adhesion complexes in the *gbp1* morphants. The Duolink PLA strategy was developed to investigate protein–protein interactions (Fig. 4a). Compared with the Tg(*tg*:GFP) embryos of the WT embryos at 4 dpf, the interaction between β-catenin and E-cadherin, two key proteins in the complex of cell–cell adhesion junctions, were significantly increased in the TP of the Tg(*tg*:GFP) embryos of the *gbp1* morphants using the Duolink PLA strategy (Fig. 4b and c).

Furthermore, this study investigated whether decreased cell–cell connection restored the abnormal morphogenesis of the thyroid gland observed in *gbp1* morphants. The use of latrunculin B (LatB), an actin polymerization inhibitor that could disturb the formation of the adhesion complex, restored the morphology of the TP in the embryos of *gbp1* morphants to normal at 5 dpf by *tg* expression (Fig. 4d). Calculation indexes including TP surface area (Fig. 4e), angles between the posterior two strands (Fig. 4f), and length to width ratio (Fig. 4g), were all improved in the embryos of *gbp1* morphants at 5 dpf after treatment with LatB. Moreover, the expression of *tshba* at 5 dpf (Fig. 4h), follicles formed at 6 dpf (Fig. 4i and j), and thyroxine-producing follicles at 9 dpf (Fig. 4k and l) were restored in the embryos of *gbp1* morphants treated with LatB, which further indicated that compromised cellular dissociation might underlie the pathological thyroid development in *gbp1*-deficient embryos.

### Enforced cellular connection impeded normal TP morphogenesis

As previously mentioned, this study found that GBP1 inhibited the expression of adhesion complexes in TPC1 cells. To summarize, *GBP1* may play a role in thyroid morphogenesis by suppressing adhesion complex formation and promoting migratory behavior. To determine whether decreased membrane β-catenin-mediated adhesion complex formation is an important contributor to the enhanced migration capacity triggered by *hGBP1* overexpression, this study artificially overexpressed membrane-anchored β-catenin. This was prepared by adding a carboxyl-terminal CAAX motif to the C-terminal of β-catenin (β-catenin-CAAX). β-catenin-CAAX overexpression compromised the role of *hGBP1* overexpression in elevating migration-related proteins such as VIM, IQGAP1, and MMP2 (Fig. S6A). Artificially overexpressing membrane-anchored β-catenin in thyroid cells under the *tg* promoter also triggered the development of condensed TP at 5-dpf embryos, as observed by *tg* expression (Fig. S6B–E). Affected thyroid function by artificially overexpressing membrane-anchored β-catenin in zebrafish embryos was verified as reduced follicle numbers (Fig. S6F) and thyroxine-containing follicles (Fig. S6G). Thus, these findings suggest that suppression of cellular dissociation impedes normal TP morphogenesis.

## DISCUSSION

Primary CH can be divided into thyroid dysgenesis (TD) and thyroid dyshormonogenesis. TD is the most frequent form of primary and permanent CH, comprising approximately 80–85% of cases in the White population. However, approximately 50% of CH cases in China have resulted in thyroid dyshormonogenesis, caused by pathogenic variants of genes that play key roles in thyroid hormone biosynthesis. Thyroid dysgenesis is a consequence of abnormal thyroid gland organogenesis and includes athyreosis (20–30%), ectopic thyroid gland (50–60%), and hypoplasia (5%). At present, only a small number of TD cases have been explained by molecular defects in *TSHR* and thyroid transcription factors, such as *NKX2-1*, *FOXE1*, *PAX8,* and *HHEX*, which are restrictedly expressed in thyroid tissues during the early developmental stages of thyroid budding and migration, and play critical roles in thyroid organogenesis [37]. A two-hit model has also been recently proposed, which comprised a germline pathogenic variation (first hit) and a somatic pathogenic variation or epigenetic modification (second hit) of genes, involved in the pathogenesis of patients with TD [10]. In this study, four nonsynonymous variations in *GBP1* were identified from three patients in three pedigrees via ES in 98 Chinese patients with CH. The transmission patterns of *GBP1* in pedigree 79 were consistent with autosomal recessive inheritance; however, the heterozygous *GBP1* variation did not explain the pathogenesis of CH in the other two pedigrees. This is because the heterozygous *GBP1* variation was inherited from either the euthyroid mother or father of the probands. By combining methylation-specific PCR and pyrosequencing, this study found that the CpG site (cg12054698) of *GBP1* was hypermethylated in the genomic DNA isolated from the two probands CH 90 and CH 168 compared with their euthyroid parents with the heterozygous *GBP1* variation. Studies have revealed the transgenerational epigenetic inheritance from one organismal generation to the next. However, the methylation levels of the CpG site were low in both parental samples of CH 90 and CH 168 examined. As we know that the epigenome is susceptible to environmental factors, including exogenous chemicals and diet, and also affected by endogenous molecules and pathophysiological conditions. We suspect that possible environmental factors might influence the methylation level of the CpG site during pregnancy. This is a subject worthy of in-depth study in the future. Although inverse correlations between the CpG site and the expression levels of *GBP1* were found among normal thyroid tissues, the expression of *GBP1* in these patients were not examined. It is also an important issue that needs to be further studied.

Collectively, these findings suggested that a two-hit model contributed to the penetrance of CH in the two pedigrees. Notably, the *zfgbp1* knockdown in zebrafish by morpholino increased the expression of thyroid stimulating hormone subunit beta a (*tshba*), one of the most sensitive markers for hypothyroidism diagnosis, and also induced a condensed abnormal thyroid morphology at 5-dpf embryos. Moreover, the follicle numbers in the thyroid tissues at 6-dpf embryos and thyroxine-positive units at 9-dpf embryos were dramatically reduced in the embryos of *gbp1* morphants. These findings support the hypothesis that hypothyroidism develops in *gbp1* deficiency zebrafish. It should be noted that the highly adhesive thyroid morphology, the main feature observed in *gbp1*-deficient embryos, was specifically restored with combined WT *hGBP1* mRNA overexpression except for the combined mutated *hGBP1* (p.H150Y, p.L187P) in zebrafish embryos. These results support the pathogenic properties of mutated *GBP1* identified in patients with CH.

AJs are protein complexes found at cell–cell junctions of epithelial and endothelial tissues that are connected to the actin cytoskeleton of adjacent cells, and play critical roles in the migration of collective cells [13]. Maintenance of cell–cell adhesion is indispensable for connective migration and relocalization of TP in mice. Through time-lapse in vivo observation of TP morphogenesis in zebrafish, this study confirmed cell dissociation followed by the formation of individual follicles. Cellular dissociation is indispensable for later TP morphogenesis, as enforced cell adhesion also impedes the formation of functional follicles in zebrafish embryos. These findings that *GBP1* decreased the adhesion complex formation in the developmental thyroid epithelial cells are consistent with previous findings that GBP1 promotes the migration and invasion of several types of cancer, such as lung adenocarcinoma [38] and glioblastoma multiforme [39]. This study also found that the deficiency of *GBP1* increased the expression of E-cadherin and β-catenin in the membrane of thyrocytes to form the adhesion complex, leading to highly adhesive thyroid morphology and hypothyroidism in *gbp1*-deficient embryos. Moreover, the abnormal morphology of the TP induced by *gbp1* deficiency was restored to normal at 5-dpf embryos of zebrafish treated with LatB, which could disturb the formation of adhesion complexes. These data suggest that the deficiency of *GBP1* induced hypothyroidism and thyroid dysgenesis might be mediated by promoting the formation of adhesion complexes in the thyroid primordium.

In summary, in this study, we found that *GBP1* variations are a potential cause of thyroid dysgenesis and congenital hypothyroidism in patients. Using a zebrafish model organism, we demonstrated that GBP1 regulated TP morphogenesis and induced hypothyroidism might be mediated by suppression of β-catenin-dependent adhesion complex formation. Moreover, the misregulated cellular adhesion status during TP morphogenesis could be a significant cause of congenital hypothyroidism.

## Supplementary information

Supplementary Method

Supplementary Movie S1

Supplementary Movie S2

## Data Availability

All data and materials used in this study are available upon request.
