# Supplementary material for: Upregulation of GBP1 in thyroid primordium is required for developmental thyroid morphogenesis
Source: Genet Med. 2021 Jun 30;23(10):1944–51. doi: 10.1038/s41436-021-01237-3 (PMC8486662; doi:10.1038/s41436-021-01237-3)
Supplement: Supplementary file 1 — Supplementary Method [file 41436_2021_1237_MOESM1_ESM.pdf]

Supplementary Methods

Whole exome sequencing (WES), sequence data processing, and quality control

Genomic DNA was extracted from peripheral blood using the Quick Gene DNA Whole Blood Kit L (Kurabo, Japan) according to the manufacturer's protocol. Whole-exome libraries were identified using approximately 1–3 μg of genomic DNA from 98 CH patients. The DNA was sheared to a range of 200–300 bp using the Covaris S2 Instrument and then phosphorylated, adenylated, and ligated to barcoded sequencing adapters using the KAPA HyperPrep Kit (Roche). The ligated DNA was size-selected for lengths between 300 and 400 bp and subjected to exonic hybrid capture according to the Roche SeqCap EZ Library SR User's Guide. The quality and concentration of the libraries were assessed with the PE LabChip GXII Touch using the MAN_CLS140145 DNA 1 K Chip. Paired-end libraries were then sequenced with the Illumina HiSeq3000 with 150 bp paired-end reads, achieving an average of ~100X depth.

The whole-exome sequence data processing and analysis of 98 patients with CH were performed according to our previous studies. First, Illumina reads were aligned to the reference human genome (hg19) using the Burrows-Wheeler Aligner (BWA) [1,2]. Only those uniquely mapped single reads or confidently mapped paired-end reads were retained after alignment. Thereafter, variations, including single nucleotide variants (SNVs) and insertions/deletions (indels), were realigned with the Genome Analysis Toolkit (GATK) software version 2.7-2. Called variations from the 98 patients with CH were filtered using the following criteria: (i) only nucleotides with a Phred quality of 50 or greater; (ii) only reads with a ratio of mapping quality equal to 0 (MQ0) and a depth less than 0.5; (iii) only nucleotides with MQ greater than 30; (iv) only variants with base quality more than 30; (v) a minimum of 5× coverage in the variants; (vi) variants with allelic heterozygosity greater than 20% were considered; and (vii) variants supported by both strands were considered. All called variants from the 98 patients with CH were filtered using the dbSNP database build 131, 132, 135, and the exome sequencing data of 170 normal individuals from our sequencing center was performed

using ESP6500, 1000 Genome Eastern Asian (EAS), ExAC EAS.

Bisulfite modification and methylation-specific PCR

DNA and MSP bisulfite modification were performed according to the manufacturer's protocol (Qiagen, 59824 and 59305). Bisulfite-modified DNA was amplified using the methylation-specific PCR primers listed in Table S2.

Pyrosequencing

The bisulfite-converted DNA was amplified using Platinum Taq DNA Polymerase (Thermo Fisher Scientific, Inc.) according to the manufacturer's protocol. PCR and pyrosequencing primers were designed by the PyroMark Assay Design 2.0. The forward and reverse primer sequences were 5'-biotin- TTTTGGAGTTAGAGA GAAAGTTGTT-3' and 5'-CAATAAAAAAATCCCAACCCTACA-3'. PCR testing was carried out at 95°C for 3 minutes, followed by 40 cycles (94°C for 30 s, 56°C for 30 s, and 72°C for 1 min, with a final extension of 72°C for 10 min). Agarose gel electrophoresis was used to confirm the quality of PCR products. Biotinylated PCR products were immobilized on streptavidin-coated Sepharose beads (GE Healthcare). The pyrosequencing primer was 5'-TCCCAACCCTACAAC-3'. Pyrosequencing was performed using PyroGold PyroMark Q96 reagents in the PyroMark Q96 ID (Qiagen, Inc.). The percentage of each CpG site was generated automatically using the PyroMark Q96 software (version1 0.6).

Global 5-mC DNA methylation quantification

The concentration of 5-methylcytosine (5-mC) in genomic DNA was quantified using 5-mC DNA ELISA kit (Zymo Research, USA) according to manufacturer's instructions. Briefly, standard curve was generated by mixing fully methylated DNA (100% M) and fully unmethylated DNA (100% U). The genomic DNA were denatured at 98°C for 5 min, and then added to 96-well microtiter plate covered with foil. After blocking, antibody dilution and color development, the absorbance reading was measured at 450 nm using ELISA plate reader. Triplicates were performed to each sample to validate the results.

Zebrafish husbandry

The methods were carried out in accordance with the approved guidelines. Transgenic *Tg(flk1: GFP)* [3] was used as previously described. *Tg(tg: GFP)* was obtained from the China Zebrafish Resource Center. *Tg(tg:mCherry)*, of which carboxyl-terminal CAAX motif fused with mCherry expressed under *tg* promoter, was created in this study.

Cell culture and reagents

TPC1 and Nthy-Ori3 cells were obtained from ATCC cultured in RPMI-1640 medium supplemented with 10% fetal bovine serum, 100 IU/mL penicillin, and 100 μg/mL streptomycin (Gibco). Mycoplasma contamination were tested routinely. Retroviral plasmids encoding WT *hGBP1* were packed in 293T cells and stably transduced into TPC1 cells using puromycin (1 μg/mL; Sigma) selection. For cell transfection, Effectene Transfection Reagent (QIAGEN, 1012829) was used according to the manufacturer's instructions. siRNA targeting human *GBP1* (Santa Cruz, sc-72088) was transfected using Lipofectamine™ RNAi MAX Transfection Reagent (Invitrogen, 13778030).

Chemical treatment on zebrafish embryos

Zebrafish embryos were incubated in E3 medium containing Latrunculin B (0. 2 ng/ml, Abcam, ab144291) from 48 hpf until collection time.

Constructs

Human *GBP1* were generated from cDNA of open reading frame sources through PCR. This was done with a Flag tag sequence added in the forward primer and ligated into mammalian expression plasmids pCS2+ or retroviral vector plasmids pMSCV-GFP (Addgene, 21654). The mutated *GBP1* were constructed using PCR-based mutagenesis. pN1-CTNNB1-CAAX was cloned by adding a GFP-CAAX (Addgene, 113020) sequence in the C-terminal of CTNNB1. Tol2 (*tg*:*zfgbp1*) were cloned by replacing the mCherry in Tol2 (*tg*:mCherry) with *zfgbp1*.

Morpholinos, mRNA synthesis, and microinjection

GBP1 MO^{atg} (5′- *CATGTTGGTTCTTTCTAGCACTTCC*-3′) and standard control morpholino were purchased from GeneTools. mMACHINE Kit (Ambion) was used for mRNA transcription. For transposon containing vector overexpression in zebrafish, 30 ng/μL transposase mRNA was co-injected.

Whole-mount *in situ* hybridization (WISH)

Whole-mount *in situ* hybridization was performed as previously described [4]. Antisense probe for *tg* was transcribed in vitro using T7 transcription kit (Roche) from plasmid containing zebrafish *thyroglobulin*. Photos were taken by Nikon SMZ25 microscope.

Zebrafish whole-mount immunofluorescence assay

In brief, embryos were fixed in 4% PFA overnight at 4 °C and then were dehydrated with methanol at -20 °C. Then the dehydrated embryos were washed three times with PBST and following Proteinase K treatment. The embryos were refixed by 4% PFA at room temperature for 20 min and washed three times with PBST. After blocking 1 hour at room temperature with blocking solution (2mg/ml BSA+10%FBS+0.3% Triton-X100+1% DMSO in PBST), antibodies were added in blocking solution, and incubate at 4 °C overnight. The samples were then washed three times with PBST and incubated with second antibody for two hours. Finally, after washing three times with PBST and the embryos were captured by confocal microscope. TUNEL staining was performed as previously described (61). pH3 and immunofluorescence staining were carried out using the antibodies pH3 (Santa Cruz Biotechnology; sc-374669), anti-Thyroxine (Abcam, ab30833).

Confocal microscopy

Live embryos were anesthetized and mounted on dishes with 1% low-melting agarose. Confocal images were captured by a Nikon A1 confocal laser microscope. The analysis of the images was carried out by Nikon confocal software and Image J.

Frozen section immunofluorescence and Duolink assays

Zebrafish embryos at indicated times were fixed with 4% formalin overnight. After washed twice in PBS, samples were cryopreserved in a sucrose gradient, embedded in O.C.T. compound (Leica), and frozen at −80°C. 7 micrometer cryosections were collected on slides. After permeabilization with 0.01% Triton X-100, Duolink assays (Sigma) were performed as the manufacturer's recommended protocol: blocking, primary antibody reaction, positive and negative probe reaction, ligation, polymerization and amplification. Antibodies used: anti-GBP1 (Sigma-Aldrich, SAB4501133), anti-β-catenin (Sigma-Aldrich, C7082), anti-E-cadherin (Sigma-Aldrich, HPA004812).

qPCR

After treatment with DNAaseI (Ambion), RNA was prepared using Trizol reagent (Invitrogen, H10522) and then subjected to cDNA synthesis using a cDNA synthesis kit (ABI). Real-time PCR was performed using a Fast Start Universal SYBR® Green Master Rox probe (Roche Applied Science, 13800300) and Mastercycler thermal cycler (Eppendorf, 22331). Primers used are shown in Table S2.

Cell immunofluorescence assay

Cell immunofluorescence was carried as previously described [5]. Cells grown to 60% confluence on coverslips were first fixed and blocked, followed by incubation with primary antibodies, including: mouse anti-FLAG (Sigma-Aldrich, F7425), mouse anti-β-catenin (Sigma-Aldrich, C7082), rabbit anti-GBP1 (Sigma-Aldrich, SAB4501133). The secondary antibodies used include: goat anti-mouse Alexa Fluor 594 (Invitrogen), goat anti-mouse, Alexa Fluor 488 (Invitrogen), goat anti-rabbit, Alexa Fluor 594 (Invitrogen).

Membrane and cytosol fraction assay

Forty-eight hours after transfection, total lysates were obtained by lysing cells from a 10-cm dish in 1 mL RIPA buffer on ice. The cytosolic and membrane fractions were separated by sucrose gradient fractionation, as previously described in the protocol by Abcam (http://www.abcam.com/ps/pdf/protocols/sub cellular fractionation.pdf).

Briefly, cells were lysed with a subcellular fractionation buffer and cell lysates were centrifuged. The supernatant was then centrifuged at a higher speed (100,000 *g*). The supernatant, after ultra-centrifugation, is the cytoplasmic fraction. The membrane fraction is obtained by washing the pellet with fractionation buffer and resuspended in nuclear buffer.

Tissue immunohistochemical (IHC) staining assays

IHC staining was performed as described in our previous study [6]. In brief, paraffin sections of 5 μm thickness were prepared. The sections were deparaffinized, treated with 3% H2O2 for 10 min, autoclaved in 10mM citric sodium (pH 6.0) for 30 min for heat-induced antigen retrieval, and then incubated with primary antibodies at 4 °C overnight, followed by incubation with biotinylated secondary antibody for 1 h at room temperature. Finally, 3,3-diaminobenzidine tetrahydrochloride was used as coloring reagent, and hematoxylin was used as a counterstain for nuclei. The stained fields were photographed using Olympus camera. Quantification of IHC staining was based upon the staining intensity (I score: negative, 0; weak, 1; moderate, 2; and intense, 3) and the percentage of positive stained cells (P score: 0–5%, score of 0; 6–35%, score of 1; 36–70%, score of 2; and >70%, score of 3) to obtain a final score (Q score = I score × P score). Sample with Q score of ≥ 4 were considered to be high expression, and < 4 were considered to be low expression. Two senior pathologists performed the scorings independently in a blinded manner.

Supplementary Figures

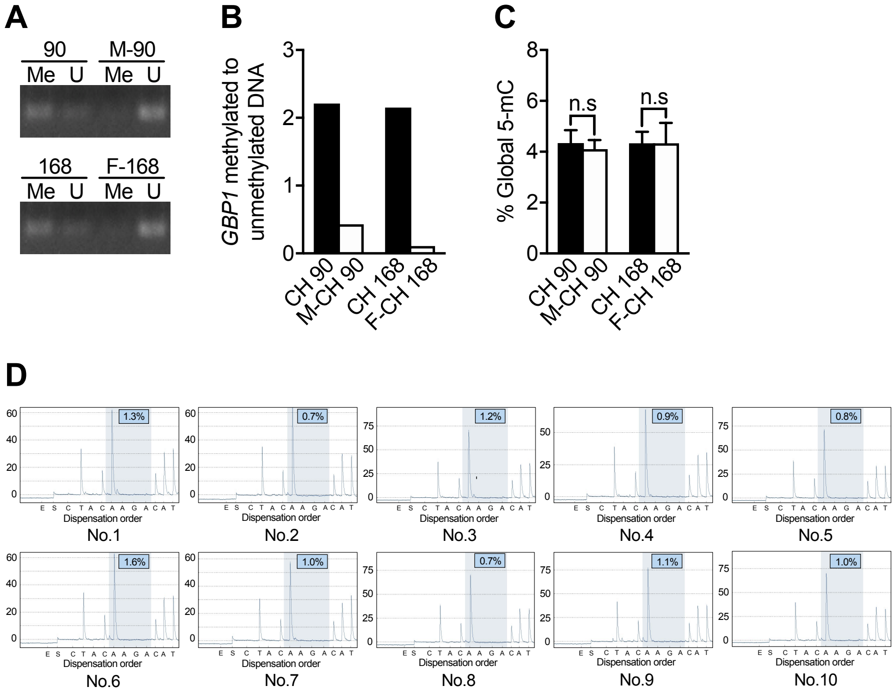

Figure S1. Methylation status of the CpG site (cg12054698) examined in age, sex and district paired control samples. (A) Methylation levels of the CpG site (cg12054698) in *GBP1* analyzed in probands and the corresponding euthyroid parents by methylation-specific PCR. "Me" indicates PCR primers designed against the methylated products, and "U" indicates primers designed against the unmethylated products. **(B)** The ratios of methylated to unmethylated products for the CpG site (cg12054698) of *GBP1* in proband 90, proband 168, and their corresponding euthyroid parents via grey value scan. **(C)** Global methylation levels in CH 90, CH 168 and their mother and father respectively were examined by 5-mC DNA ELISA kit. **(D)** Methylation level of the CpG site (cg12054698) among 10 control samples examined by pyrosequencing. n.s = not significant; (Student's t-test).

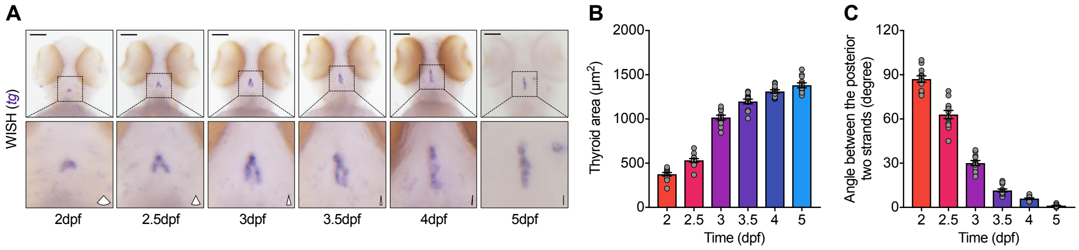

Figure S2. Zebrafish thyroid development underwent dynamic morphological changes. (A) WISH assessment of *tg* expression in 2 dpf to 5 dpf zebrafish. Right lower corner are the schematic diagrams show the angles between the posterior strands of TP. Bars = 50μm. **(B)** Surface of TP surface area calculated by *tg* expression from 2 dpf to 5 dpf. N = 12. **(C)** Angles between the posterior strands calculated by *tg* expression pattern from 2 dpf to 5 dpf. N = 12.

A

GBP1 protein

```
human      1    MASEIHMTGPMCLIENTNGRLMANPEALKILSAITQPMVVVAIVGLYRTGKSYLMNKLAG  60
                M+    M  P+CL+EN NG L    +A+  LS I +P+VVV++VGLYRTGKSYLMN+LAG
zebrafish  1    MSKPTMMPSPICLVENVNGSLSICKDAIDFLSNINEPVVVVSVVGLYRTGKSYLMNRLAG  60
human      61   KKKGFSLGSTVQSHTKGIWMWCVPHPKKPGHILVLLDTEGLGDVEKGDNQNDSWIFALAV  120
                ++ GF+LG+T++S TKGIWMWCVPHPKK GH LVLLDTEGLGDV+KGD++ND WIF LAV
zebrafish  61   QQTGFALGNTIESKTKGIWMWCVPHPKKQGHTLVLLDTEGLGDVDKGDSKNDGWIFCLAV  120

human      121  LLSSTFVYNSIGTINQQAMDQLYYVTELTHRIRSKSSPDENENEVEDSADFVSFFPDFVW  180
                LLSST VYNS GTI+  A+++L+Y+TELT +I+ +++   +E + EDS  FV FFP F W
zebrafish  121  LLSSTLVYNSRGTIDNSALEKLHYITELTEQIKIRTAEAADEEDEEDSK-FVLFFPSFTW  179

human      181  TLRDFSLDLEADGQPLTPDEYLTYSLKLKKGTSQKDETFNLPRLCIRKFFPKKKCFVFDR  240
                 +RDF+LDL  DG+ +  D+YL ++L+L+KG ++K   +NLPR CIR +FP +KCFVF
zebrafish  180  VVRDFTLDLVIDGKNVNEDQYLDFALQLRKGNNKKALEYNLPRQCIRNYFPSRKCFVFPS  239

human      241  PVHRRKLAQLEKLQDEELDPEFVQQVADFCSYIFSNSKTKTLSGGIQVNGPRLESLVLTY  300
                P    K+ QLE LQ+++L P+F++    FC +IF NS  KTL GG +V G  L  L   Y
zebrafish  240  PASPEKMKQLESLQEKDLVPDFLEVTGRFCEHIFRNSVVKTLKGGHKVTGKLLGHLAKIY  299

human      301  VNAISSGDLPCMENAVLALAQIENSAAVQKAIAHYEQQMGQKVQLPTETLQELLDLHRDS  360
                V+ ISSG +PC++NAV+ALA +EN  AV++A   YE+ M +  +     + E+   H+
zebrafish  300  VDTISSGKVPCLDNAVVALANLENKCAVEEAFKVYERGMEEVKKTFPVNVDEISSEHQRF  359

human      361  EREAIEVFIRSSFKDVDHLFQKELAAQLEKKRDDFCKQNQEASSDRCSALLQVIFSPLEE  420
                   A   F++ SFKD    +  +L  +++        + N  AS  +C  LL+ +FS + +
zebrafish  360  SSLANAEFMKRSFKDEKQEYINQLKEKIDVYYGCLLEDNGLASKKKCEDLLKDLFSEMNK  419

human      421  EVKAGIYSKPGGYRLFVQKLQDLKKKYYEEPRKGIQAEEILQTYLKSKESMTDAILQTDQ  480
                +++ G+YS+PGGY L+ +    + +KY  EP KG+ AE +L  +L  + +  ++IL TD
zebrafish  420  KLQDGVYSQPGGYELYCKDRDVIVEKYRREPNKGVLAEAVLHEFLNERGAEANSILYTDT  479

human      481  TLTEKEKEIEVERVKAESAQASAKMLQEMQRKNEQMMEQKERSYQEHLKQLTEKMENDRV  540
                 LTE +K+I+ E+ +    Q    +  QE + ++E+MM  ++    + +KQ+ EK E +R
zebrafish  480  KLTENDKKIQEEKERNALMQQKCEEEQEKRIESERMMAAEKVRQDDQIKQMQEKFEKERE  539

human      541  QLLKEQERTLALKLQEQEQLLKEGFQKESRIMKNEIQDLQ  580
                Q+ +E E+ +  KL+EQE++L +GF++ + ++  EI DL+
zebrafish  540  QMQQETEKAIDSKLKEQEEMLNKGFKERADLLSEEINDLK  579
```

B

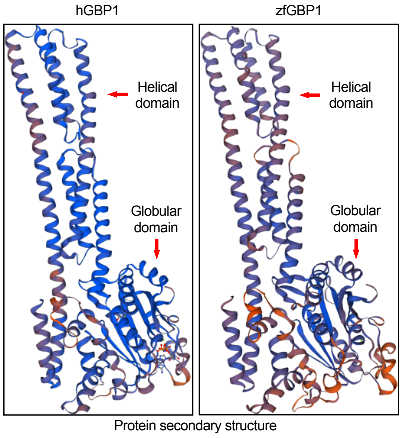

C

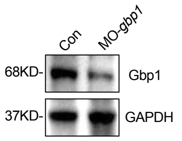

Figure S3. Protein structure conservation between human and zebrafish GBP1. (A) The conservation of the amino acid sequence between human and zebrafish GBP1. **(B)** The secondary structure of human GBP1 (hGBP1) and zebrafish GBP1 (zfGbp1) protein. Both harbor a N-terminal globular domain and a C-terminal helical domain. **(C)** The knock-down efficiency of *gbp1* was examined by western blot.

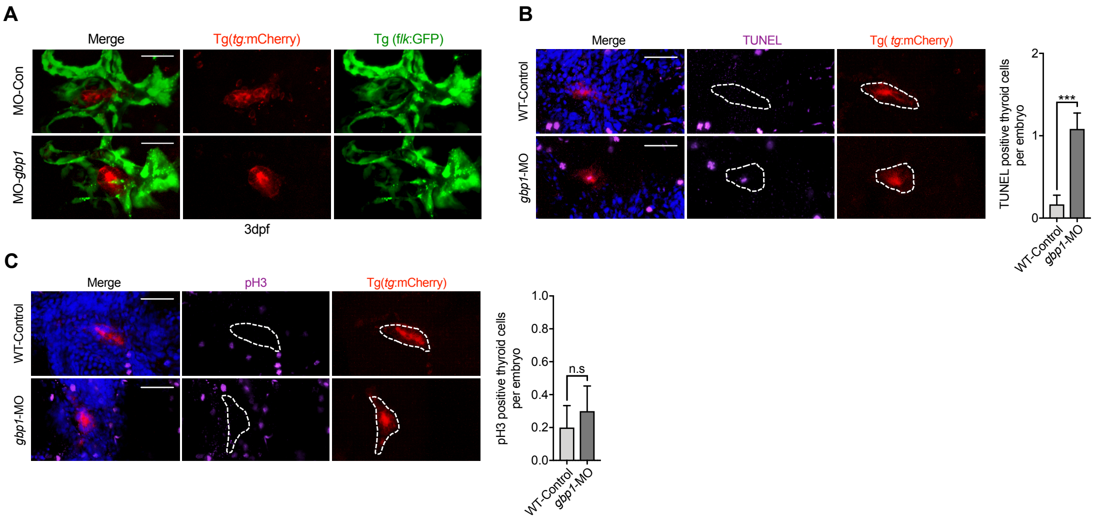

Figure S4. The effect of *gbp1* knockdown on thyroid follicular cell proliferation and apoptosis in zebrafish embryos at 3 dpf. (A) Confocal examination of thyroid development in WT and *gbp1* morphants using Tg (*flk*:GFP), a marker specifically expressed in endothelia and Tg (*tg*:mCherry) double transgenic lines. Bars = 50μm. N=10. **(B)** Thyrocyte apoptosis in WT and *gbp1* knockdown Tg:(*tg*:mCherry) transgenic zebrafish embryos, detected by TUNEL staining. Bars = 50μm. N=10. **(C)** Thyrocyte proliferation in WT and *gbp1* knockdown *Tg:(tg*:mCherry*)* transgenic zebrafish embryos, detected by pH3 staining. Bars = 50μm. N=10. n.s = not significant; $^{***}P < 0.001$; (Student's t-test).

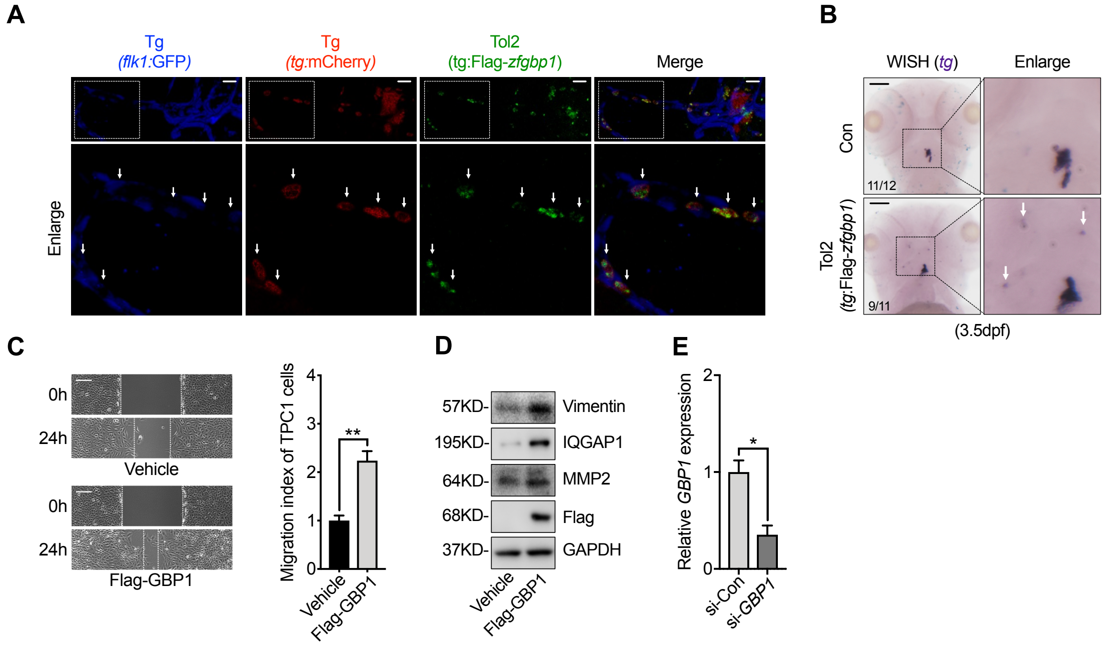

Figure S5. *GBP1* overexpression promotes the migration of thyroid cells. (A) Tg(*flk*:GFP) and Tg(*tg*:mCherry) double transgenic lines used for analyzing the effect of overexpressing *flag-zfGbp1* in thyroid cells. Lower are the enlarged ones. The arrows indicate the ectopic thyroid cells migrated along vascular overexpressing Flag-*zfGbp1* (Flag and mCherry double positive). Bars = 20μm. **(B)** As detected by WISH, ectopic thyroid cells were observed on the embryos overexpressing Tol2 (*tg*:*Flag-zfGbp1*) plasmids, but not on those overexpressing Tol2(*tg*:*Flag-Control*) vector plasmids. Right are the enlarged ones. White arrows indicate ectopic thyroid cells positive for *tg* signal. Bars = 50μm. **(C)** The migration capacity of TPC1 cells after being stably transfected with WT-hGBP1 plasmid were measured by scratch assays. Bars = 100μm. **(D)** Western blot examination of migration related proteins expression with Flag-*GBP1* overexpression in TPC1 cells. **(E)** RT-PCR examination of *GBP1* levels in TPC1 cells with si-RNA mediated knock down. $^{*}P < 0.05$; $^{**}P < 0.01$; (Student's t-test).

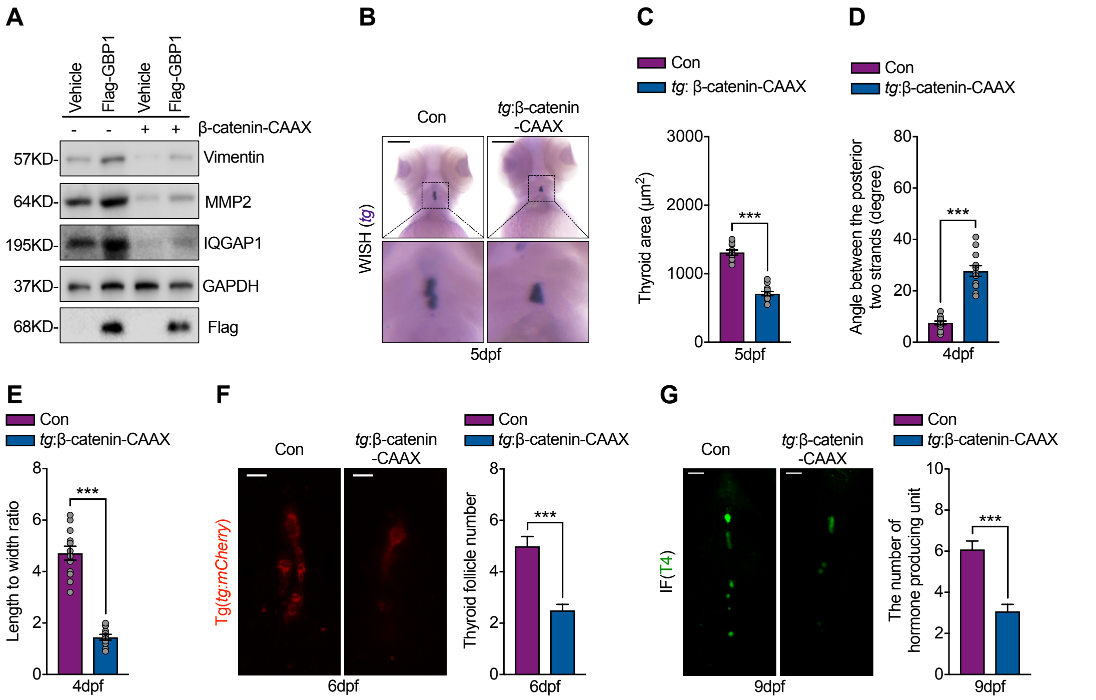

Figure S6. Enforced membrane adhesion on TP growth. (A) Expression of migration related proteins in TPC1 cells with *β-catenin-CAAX* plasmid overexpression. **(B)** Representative images show the expression pattern of *tg* in WT control and *tg*:β-*catenin-CAAX* overexpression embryos. Bars = 50μm. **(C-E)** Statistical calculation of surface area (C), angles between posterior strands (D) and length to width ratio (E) shown in (B). N = 12. **(F)** Thyroid follicles formed in WT control and *tg*:*β-catenin-CAAX* overexpression embryos and statistical assessment of the data. Bars = 20μm. **(G)** T4 containing follicles formed in WT control and *tg*:*β-catenin-CAAX* overexpression embryos and statistical assessment of the data. Bars = 20μm. N = 12. $^{***}P < 0.001$ (Student's t-test).

Table S1. List of *GBP1* variants identified in this cohort including ACMG/AMP criteria [7].

Individual	Types of variant	Nucleotide alteration (NM_002053.2)	Protein alteration (NP_002044.2)	Genomic position hg19	Zygosity	Exon number	Inheritance	CADD score[8]	SIFT[9]	ACMG/AMP[7]	ACMG/AMP criteria[7]
CH 90	NS	c.58C>T	p.(Arg20Ter)	89528860	het	1	maternal	29.2	-	P	pvs1 ps3 pm1
CH 79	FS	c.1006dup	p.(Glu336Glyfs Ter26)	89522685	het	6	maternal	-	Deleterious	P	pvs1 ps3 pp3 pm4
CH 79	MS	c.448C>T	p.(His150Tyr)	89524707	het	4	paternal	15.76	Deleterious	LP	ps3 pm1
CH168	MS	c.560T>C	p.(Leu187Pro)	89524595	het	4	paternal	22.7	Deleterious	LP	pm1 pp3 ps3

Table S2. Sequence of PCR primers

Name	Primer sequence
HU-*GAPDH*-QPCR-FP	5'-GAAGGTGAAGGTCGGAGTC-3'
HU-*GAPDH*-QPCR-RP	5'-GAAGATGGTGATGGGATTTC-3'
HU-*GBP1*-QPCR-FP	5'-AGGCATCCAGGTCAACGG-3'
HU-*GBP1*-QPCR-RP	5'-AGTTCTCTATCTGGGCCAAGG-3'
GBP1-Me-FP:	5'-ATGAGGAAATTTTAGTTTTATAATTTC-3'
GBP1-Me-RP:	5'-TTCTATATCTCTCACTAAATCCC-3'
GBP1-U-FP:	5'-GAGGAAATTTTAGTTTTATAATTTTGG-3'
GBP1-U-RP:	5'-TTCTATATCTCTCACTAAATCCC-3'
Zf-*tshba*-FP:	5′- GTTATTGGCATGCTGGGACT-3′
Zf-*tshba*-RP:	5′- GTGCATCCCCTCTGAACAAT-3′

References

1. Chu X, Pan CM, Zhao SX, et al. A genome-wide association study identifies two new risk loci for Graves' disease. Nat Genet. 2011;43(9):897-901.
2. Zhang LL, Kan M, Zhang MM, et al. Multiregion sequencing reveals the intratumor heterogeneity of driver mutations in TP53-driven non-small cell lung cancer. Int J Cancer. 2017;140(1):103-108.
3. Beis D, Bartman T, Jin SW, et al. Genetic and cellular analyses of zebrafish atrioventricular cushion and valve development. Development. 2005;132(18):4193-4204.
4. Hinits Y, Osborn DP, Hughes SM. Differential requirements for myogenic regulatory factors distinguish medial and lateral somitic, cranial and fin muscle fibre populations. Development. 2009;136(3):403-414.
5. Yang RM, Tao J, Zhan M, et al. TAMM41 is required for heart valve differentiation via regulation of PINK-PARK2 dependent mitophagy. Cell Death Differ. 2019;26(11):2430-2446.
6. Yang R, Liang X, Wang H, et al. The RNA methyltransferase NSUN6 suppresses pancreatic cancer development by regulating cell proliferation. EBioMedicine. 2021;63:103195.
7. Richards S, Aziz N, Bale S, et al. Standards and guidelines for the interpretation of sequence variants: a joint consensus recommendation of the American College of Medical Genetics and Genomics and the Association for Molecular Pathology. Genet Med. 2015;17(5):405-424.
8. Rentzsch P, Witten D, Cooper GM, Shendure J, Kircher M. CADD: predicting the deleteriousness of variants throughout the human genome. Nucleic Acids Res. 2019;47(D1):D886-D894.
9. Pollard KS, Hubisz MJ, Rosenbloom KR, Siepel A. Detection of nonneutral substitution rates on mammalian phylogenies. Genome Res. 2010;20(1):110-121.
